# WNT5A Inhibits Metastasis and Alters Splicing of Cd44 in Breast Cancer Cells

**DOI:** 10.1371/journal.pone.0058329

**Published:** 2013-03-06

**Authors:** Wen Jiang, David K. Crossman, Elizabeth H. Mitchell, Philip Sohn, Michael R. Crowley, Rosa Serra

**Affiliations:** 1 Department of Cell, Developmental, and Integrative Biology, University of Alabama at Birmingham, Birmingham, Alabama, United States of America; 2 Department of Genetics, University of Alabama at Birmingham, Birmingham, Alabama, United States of America; Case Western Reserve University, United States of America

## Abstract

Wnt5a is a non-canonical signaling Wnt. Low expression of WNT5A is correlated with poor prognosis in breast cancer patients. The highly invasive breast cancer cell lines, MDA-MB-231 and 4T1, express very low levels of WNT5A. To determine if enhanced expression of WNT5A would affect metastatic behavior, we generated WNT5A expressing cells from the 4T1 and MDA-MB-231 parental cell lines. WNT5A expressing cells demonstrated cobblestone morphology and reduced in vitro migration relative to controls. Cell growth was not altered. Metastasis to the lung via tail vein injection was reduced in the 4T1-WNT5A expressing cells relative to 4T1-vector controls. To determine the mechanism of WNT5A action on metastasis, we performed microarray and whole-transcriptome sequence analysis (RNA-seq) to compare gene expression in 4T1-WNT5A and 4T1-vector cells. Analysis indicated highly significant alterations in expression of genes associated with cellular movement. Down-regulation of a subset of these genes, Mmp13, Nos2, Il1a, Cxcl2, and Lamb3, in WNT5A expressing cells was verified by semi-quantitative RT-PCR. Significant differences in transcript splicing were also detected in cell movement associated genes including Cd44. Cd44 is an adhesion molecule with a complex genome structure. Variable exon usage is associated with metastatic phenotype. Alternative spicing of Cd44 in WNT5A expressing cells was confirmed using RT-PCR. We conclude that WNT5A inhibits metastasis through down-regulation of multiple cell movement pathways by regulating transcript levels and splicing of key genes like Cd44.

## Introduction

The Wnt family of proteins consists of at least 19 members, that can be broadly divided into two general categories: 1) the canonical, ß-catenin pathway; and (2) the non-canonical, ß-catenin independent pathway [1], [2], [3]. While the Wnt/ß-catenin pathway has been studied extensively, less is known about the non-canonical pathways, which include Planar Cell Polarity and Wnt/Ca^+2^ signaling [4], [5]. Many canonical signaling Wnts have a clear role in breast cancer progression [2], [6]. A screen of Wnt expression in various established tumor cell lines showed that, in general, canonical Wnts were up-regulated in cancer cell lines relative to normal human mammary epithelial cells while the expression of non-canonical Wnts, including WNT5A, WNT5B and WNT16, was down-regulated [7], [8]. Previous studies have shown that loss of WNT5A is associated with early relapse of invasive breast cancer and, in a retrospective study, immunohistochemical detection of WNT5A in tumors was inversely correlated with metastasis and survival [8], [9], [10]. In contrast, it was shown that WNT5A is critical for macrophage-induced invasion of breast cancer cell lines [11], [12]. This suggests WNT5A may play different roles, which may be stage dependent or involve cues from the microenvironment (reviewed in [13]. Therefore, an in-depth understanding of the mechanism of WNT5A action in breast cancer progression and metastasis is required.

Cell movement is an integral part of metastasis. Migration is regulated by numerous chemokines, cytokines, and growth factors that in general promote cell migration by causing changes in the cytoskeletal structure and cell adhesion. When added to cells in culture, WNT5A inhibits migration in part by increasing adhesion [14], [15]. Drugs that target migration of tumor cells could be used to combat metastatic disease. Recently, a WNT5A peptide agonist, FOXY-5 was shown to inhibit breast cancer metastasis in an in vivo mouse model [16]. Although WNT5A is known to inhibit migration in breast cancer cell lines, the consequences of WNT5A expression on specific migration associated gene targets are not known.

Cell behavior is ultimately dictated by the complement of mRNAs that are expressed in the cell. In addition to generating hypotheses, global analysis of gene expression can be used as a way to phenotype cells and is now routinely used to classify breast cancer subtypes [17]. Expression microarrays are the most common method used for this type of gene analysis. More recently, ultra-high throughput sequencing has been used to analyze the entire transcriptome within a cell. The advantages of mRNA sequencing (RNA-seq) include the ability to obtain more comprehensive coverage relative to microarrays and the identification of specific splice variants of a particular gene [18], [19], [20]. This is in addition to being able to determine the presence and amount of each mRNA relative to another sequenced sample. Recently, RNA-seq has become more cost effective than microarray and analysis of the huge amounts of data generated from each experiment has become standardized making RNA-seq a feasible way to study the mechanisms of cell behavior.

An inverse correlation between malignant potential and WNT5A expression exists among several breast cancer cell lines [7], [8]. MDA-MB-231 and 4T1 breast cancer cells are highly metastatic and express little to no WNT5A. In this study, we generated cell lines that ectopically express WNT5A to study the effects of WNT5A on metastatic behavior. To determine the mechanism of WNT5A action on metastasis, we used microarray and RNA-seq analysis to determine differences in gene expression between parental and WNT5A-expressing cells. We show that expression of WNT5A regulates expression and splicing of genes associated with several migration pathways. We propose that WNT5A acts at least in part to inhibit metastasis by regulating movement of tumor cells through these changes in gene expression and transcript splicing.

## Materials and Methods

### Ethics Statement

All animal experiments were done after approval of the University of Alabama Institutional Animal Care and Use Committee, protocol number 08539 to R. Serra.

### Cell Lines

Mouse 4T1, human MDA-231, L-parental and L-WNT5A cells were obtained from ATCC. 4T1-Luc cells were obtained from Caliper Life Sciences. 4T1-Luc cells are resistant to Puromycin. Cells were grown according to the manufacturer’s instructions. Cells were imaged on an Olympus (CK40) inverted microscope using phase contrast microscopy and a magnafire digital camera.

### Production of WNT5A Expressing Stable Cell Lines

Lentivirus containing the human WNT5A cDNA (hWNTA) was generated using the pLenti6/V5 Directional TOPO Cloning Kit (Invitrogen K4955-10). Phusion High-Fidelity DNA polymerase was used to produce the blunt–end PCR product of hWNTA for cloning. Both the control vector (pLenti6/V5-GW/lacZ) and expression vector had a Blasticidin selection marker. Sequencing and restriction enzyme digestion was used to verifying the correct clones, including the orientation of the insert. Lentivirus was produced by co-transfection of the packaging and envelope plasmids (pCMV-VSV-G and psPAX2) with the pLenti6/V5-D-TOPO expression vector into 293T cells (Invitrogen). Two to three days post-transfection, hWNT5A containing and control lenti-viruses were collected and used to infect mouse 4T1 cells. One to two days later, infected cells were selected for Blasticidin resistance. Stable cell lines were expanded after 10–14 days of Blasticidin selection. Similar methods were used to generate control and WNT5A-expressing cells from mouse 4T1-Luciferase positive cells and human MDA-MB-231 cells.

### Western Blot

To detect endogenous and ectopic WNT5A, cells were lysed in NP40 lysis buffer (150 mM sodium chloride, 1.0% NP-40, 50 mM Tris, pH 8.0), protein was quantified using the Biorad Protein Assay. Proteins were separated on a 10% gel using SDS- PAGE. Protein was transferred to PVDF membrane. Monoclonal Anti-human/mouseWnt-5a Antibody (from R&D, Cat: MAB645) and ECL chemiluminescence was used to detect WNT5A protein on the blots. GAPDH (Santa Cruz, SC-20357) was used as a loading control.

### Immunofluorescent Staining

The cells were grown in chamber slides then were washed twice with PBS and fixed and permeabilized with cold methanol. Cells were rinsed with PBS three times in blocking solution (10% normal goat serum (vector Laboratories cat # S-1000) in TBST) for 30 minutes at room temperature. The cells were stained with the E-cadherin antibody (Cell Signaling cat # 4065S) at 1∶200 for 30 min. The cells were then washed three times with PBS, and then incubated with the secondary antibody (Alexa 488 conjugated goat anti-rabbit IgG, Invitrogen cat # A11008), at 1∶200 for 30 min. The samples were washed and then mounted with aqueous mounting solution.

### Growth Curve

Cells were plated in 24 well plates at 6150 cells per well. Day 1 was considered the day after plating. For counting, cells were trypsinzed with 125 ul of 0.05 M Trypsin. Trypsin was inactivated with FBS and the cells were diluted in trypan blue 1∶2 for days 1 and 3 and 1∶5 for days 5, 8, 10. The total number of cells/well was then calculated. The data was plotted as cells per well over days in culture. The experiment was repeated two times with similar results. A representative experiment is shown. Vector and 4T1-WNT5A growth curves were analyzed with the extra sum of squares F-test comparing the rate constant K of the lines of best fit using Prism 6 software.

### Migration Assay

CFDA working solution (10uM) was prepared using PBS. Cells were loaded with CFDA. The migration assay was performed using the 96 well ChemoTx plate from Neuro probe (Gaithersburg, MD) according to the manufacturer’s instructions. After incubation overnight, the top cells were removed gently with a cotton swab and the top surface of the filter was washed with media. The cells were spun down while in the microplate and migrated cells were counted using a fluorescence microplate reader. Before the start of the migration assay, the cells were serum starved overnight. Complete media with 10% FBS was placed into the bottom well as a chemoattractant. All of the migration assays were repeated at least three times.

### Conditioned Media

L-parental and L-WNT5A cells (from ATCC) were split into culture medium without G418 and the cells were grown for 4 days (approximately to confluence). The medium was removed and filter sterilized. Fresh culture medium without G418 was added to the cells and the cells were cultured for another 3 days. The medium was collected, filtered and added to the media collected the first time.

### Tail Vein Injection Assay

All animal experiments were done after approval of the University of Alabama Institutional Animal Care and Use Committee. Balb/c mice were used for the experiments. 2×10^5^ of the indicated cells was injected into the mouse tail vein using standard procedures (n = 6 vector and n = 6 WNT5A injected). At varying times after injection, luciferase imaging was performed. Mice were anesthetized and injected i.p. with luciferin (2.5 mg/0.1 ml). Fifteen minutes after injection, animals were imaged using an IVIS-100 system at the UAB Small Animal Imaging Core Facility. Data acquisition software ensured that no pixels were saturated during image collection. Light emission from animal regions (photons/sec) were measured using software provided by the vendor (Xenogen). The intensity of light emission was represented with a pseudo color scaling of the bioluminescent images. The bioluminescent images were overlaid on black and white photographs of the animals that were collected at the same time.

### Microarray

Affymetrix Mouse Exon GeneChip ST 1.0 Array interrogates over 28,000 well-annotated mouse genes with 764,000 distinct probe sets. The GeneChip analysis was carried out in the Gene Expression Shared Facility located in the Heflin Center for Genomic Sciences. The facility is equipped with the Affymetrix Complete GeneArray Instrument System. The quality of each RNA sample was determined by analysis on the 2100 Agilent Bioanalyser prior to RNA labeling. Detailed genechip analysis procedures are presented in the manufacturer’s GeneChip Expression Technical Manual (Affymetrix). RNA was isolated from two separate passages of both vector and WNT5A cells at confluency using Trizol reagent. RNA was DNAse treated to remove DNA contamination. 100 ng of total RNA from each sample was used to generate double strand cDNA by linear amplification using T7-linked random primers and reverse transcriptase. Subsequently, cRNA was generated by standard methods (Affymetrix) followed by cRNA fragmentation, end label biotinylation and preparation of hybridization cocktail. The arrays were hybridized overnight at 45°C, and then washed, stained, and scanned the next day. Gene expression levels were extracted using AGCC (Affymetrix GeneChip Command Console).

### RNA-seq

mRNA-sequencing was performed by the using an Illumina Genome Analyze IIx (GAIIx) providing up to 95 Gb of sequence information per flow cell. One of the each of the vector and WNT5A expressing samples of RNA used in the microarray screen was poly A+ selected two times and converted to cDNA. The TruSeq library generation kit was used to generate sequencing libraries as per the manufacturer’s instructions (Illumina, San Diego, CA). Library construction consisted of random fragmentation of the polyA mRNA, followed by cDNA production using random primers. The ends of the cDNA were repaired, A-tailed and adaptors were ligated for indexing during the sequencing runs (up to 12 different barcodes per lane). The cDNA libraries were quantified using qPCR in a Roche LightCycler 480 with the Kapa Biosystems kit for library quantification (Kapa Biosystems, Woburn, MA) prior to cluster generation. Clusters were generated to yield approximately 725K-825K clusters/mm^2^. Cluster density and quality was determined during the run after the first base addition parameters were assessed. Paired end 2×50 bp sequencing runs to align the cDNA sequences to the mouse mm9 reference genome were performed.

### Bioinformatics

Statistical analysis of the GeneChip experiment was conducted using the software package GeneSprings v11 (Agilent Technologies, Santa Clara, CA). Briefly, the raw GeneChip files (.CEL files) from AGCC and Expression Console were uploaded, background-subtracted, variance stabilized, and normalized with GC-RMA method. The vector control group was used as a baseline to calculate the intensity ratio/fold changes of the treated versus the control group.

For the RNA-Seq experiments, TopHat version 2.0.0 [19] was used to align RNA-Seq reads to the reference genome (mm9) using the short read aligner Bowtie (version 2.0.0.5) [21]. TopHat was also used to analyze the mapping results to identify splice junctions between exons. Cufflinks (version1.3.0) [20] was used to align reads from TopHat and assemble transcripts, estimate their abundances and test for differential expression and regulation. Cuffcompare (version 1.3.0), which is part of Cufflinks was used to compare the assembled transcripts to a reference annotation. Finally, Cuffdiff (version 1.3.0) was used with settings of False Discovery Rate 0.05, Minimum Alignment Count 10, quartile normalization and bias correction to find significant changes in transcript expression, splicing and promoter use. Splicing was further visualized using Broad’s Integrative Genomics Viewer (IGV) [22].

The above data sets were analyzed using Ingenuity Pathways Analysis (IPA) software (Ingenuity® Systems, www.ingenuity.com). The data set contained gene identifiers and corresponding expression values and was uploaded into the application. Each identifier was mapped to its corresponding object in Ingenuity’s Knowledge Base. A fold-change cutoff of ±2 was set to identify target molecules whose expression was significantly up- or down-regulated. These Network Eligible molecules were overlaid onto a global molecular network developed from information contained in Ingenuity’s Knowledge Base. Networks of Network Eligible Molecules were then algorithmically generated based on their connectivity. The Functional Analysis identified the biological functions that were most significant to the dataset. Molecules from the dataset that met the ±2 fold-change cutoff and were associated with biological functions in Ingenuity’s Knowledge Base were considered for analysis. Right-tailed Fisher’s exact test was used to calculate a p-value determining the probability that each biological function assigned to that data set is due to chance alone.

Microarray and RNA seq data were deposited into the GEO database. The accession number for the super series, which contains both microarray and RNA-seq datasets is GSE41793.

### RT-PCR

RNA was extracted from 4T1 control and WNT5A expressing cells using RNeasy Plus Mini Kit (Qiagen, USA) and resuspended in RNase-free water. cDNA was synthesized from 1 µg total RNA using a reverse transcription kit (Qiagen, USA). Semi-quantitative PCR was set up using approximately 50 ng per sample. Each sample was analyzed at the linear range of amplification, as determined by analysis at three different cycles. cDNA levels were normalized to Gapdh: Forward: 5′-ACCACAGTCCATGCCATCAC-3′, Reverse: 5′-TCC-ACC-ACC-CTG-TTGCTG-TA-3′). Other primers were as follows: hWNT5A: Forward 5′-CCGCGAGCGGGAGCGCAT, Reverse 5′-GCCACATCAGCCAGGTTGTACACC; mMmp13, Forward: 5′-TCCTGGCCACCTTCTTCTTGTTGA-3′, Reverse 5′-AGTTTGCCAGTCACCTCTAAGCCA-3′; mNos2: Forward:5′-CTGCTGGTGGTGACAAGCACATTT-3′ Reverse 5′-CGTTCTTTGCATGGATGCTGCTGA-3′; mIl1a: Forward: 5′-ACTGATGAAGCTCGTCAGGCAGAA -3′ Reverse 5′- CCCGACTTTGTTCTTTGGTGGCAA -3′; mCxcl1: Forward: 5′-TAACCAGTTCCAGCACTCCAGACT-3′ Reverse 5′-TGTTCTTGAGGTGAATCCCAGCCA-3′; mLamb3: Forward:5′-TTAGGTGCCCGGAAAGATATGCCT-3′ Reverse 5′-TCCTGTAAACTGGTTGCACTGGGA-3′.

To characterize alternative exon usage in Cd44, primers for Cd44s, Cd44v4, Cd44v6 and Cd44v9 from Hebbard et al. 2000 [23] were used. Images were quantified using integrated pixel density analysis in Photoshop.

## Results

### Generation and Characterization of WNT5A Expressing Breast Cancer Cell Lines

**Figure 1:**
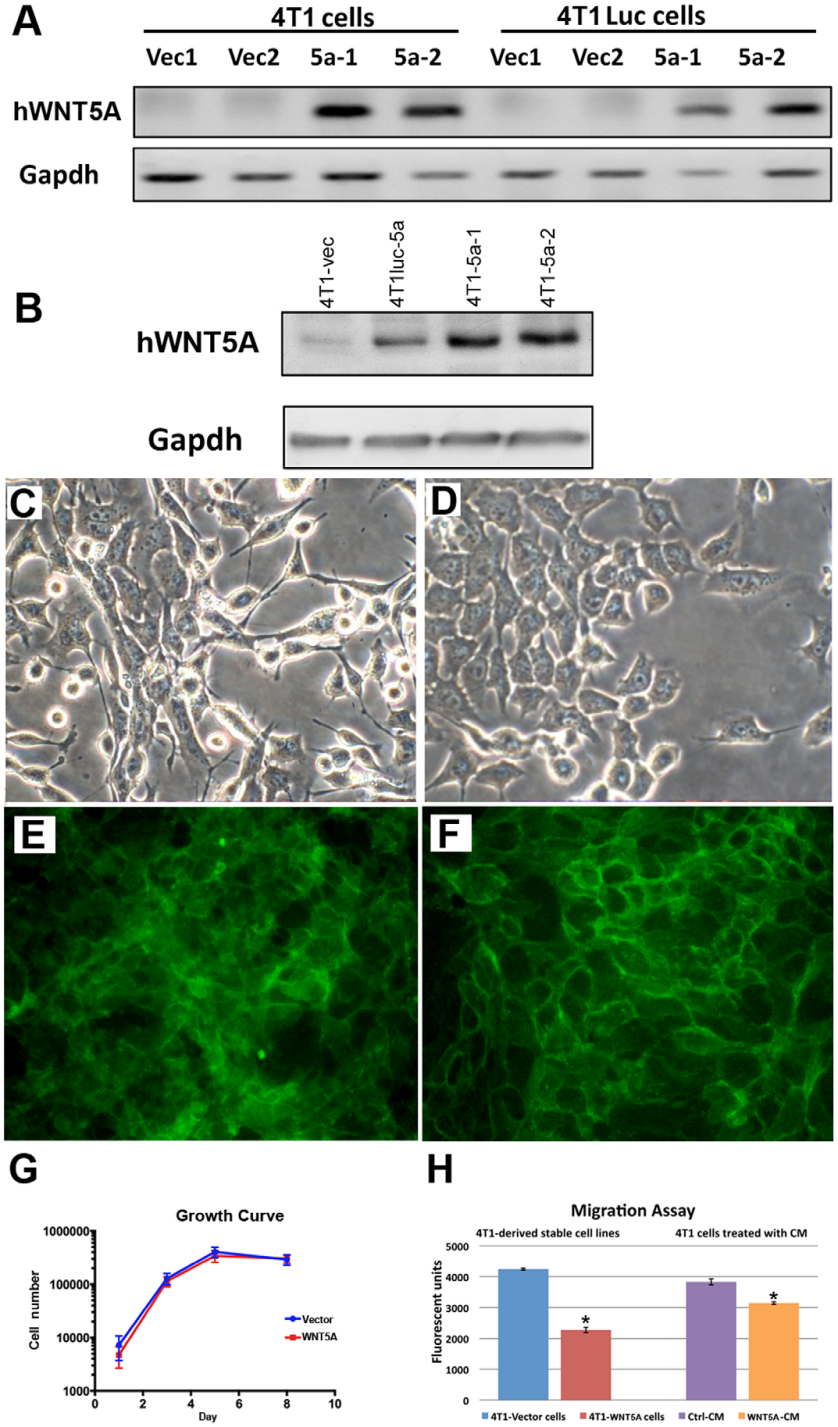
Generation and characterization of WNT5A expressing 4T1 cells. (A) RT-PCR showing expression of the human WNT5A transgene in 4T1 and 4T1-luciferase (4T1-luc) vector (vec) and WNT5A (5a) transduced cell lines. Glyceraldehyde-3-phosphate dehydrogenase (Gapdh) is used as a loading control. (B) Western blot showing expression of WNT5A in 4T1 cells transduced with vector alone (4T1-vec) and 4T1 and 4T1-luciferase cells transduced with WNT5A (4T1-5a and 4T1Luc-5a). Gapdh is used as a loading control. (C, D) Phase contrast images of 4T1-vector (C) and 4T1-WNT5A (D) cells showing changes in morphology in WNT5A expressing cells. (E, F) E-cadherin staining of 4T1-vector (E) and 4T1-WNT5A (F) cells. E-cadherin stain is green. (G) Cell counts were used to measure cell growth over 8 days. Cell growth was comparable in vector and WNT5A 4T1 cells. Extra Sum of Squares F-test, p value 0.4806. (H) Transwell migration assay was used to determine the effects of WNT5A expression on cell migration. 4T1-WNT5A cells showed reduced migration towards FBS (H, left side). Cells treated with WNT5A conditioned medium also showed reduced migration toward FBS when compared to cells treated with a control conditioned medium (H, right side). * = T-test p-value <0.05.

A highly invasive mouse breast cancer cell line that expresses low to undetectable levels of endogenous Wnt5a, 4T1, was transduced with lentiviruses that either express human WNT5A (4T1-WNT5A) or the vector alone (4T1-vector). Cells carrying the integrated virus were selected with Blasticidin. WNT5A expressing cells were also generated in a similar manner from 4T1 cells that contained a luciferase marker (4T1-WNT5A-luc and 4T1-vector-luciferase). Increased expression of WNT5A in the WNT5A-trandsduced cells was confirmed at the mRNA and protein levels by RT-PCR and Western blot (Figure 1A, B).

Next, the effects of increased WNT5A expression on the biological characteristics of the cells was examined. The morphology of the WNT5A expressing cells was altered relative to the vector only controls (Figure 1 C, D). The parental cells (not shown) and the vector only cells (Figure 1C) were refractile and spindle shape, common in highly metastatic cancer cell lines. In contrast, the WNT5A-expressing cells were flat and more cobblestone-like in appearance (Figure 1D), characteristics of normal epithelial cells. To confirm more normal cell features we stained the cells for E-cadherin (Figure 1 E, F). In 4T1-vector cells E-cadherin localization was disorganized (Figure 1E). In contrast, WNT5A expressing cells were clearly outlined with E-cadherin staining suggesting a more normal epithelial phenotype (Figure 1F). Similar staining patterns were previously reported in HB2 cells expressing varying amounts of Wnt5a [24]. The change in morphology suggested WNT5A may have an effect on growth or migration of the cells; however, we did not detect significant differences in cell growth in the 4T1-WNT5A and 4T1-vector cells over 8 days (Figure 1G). We then used a transwell assay to compare migration of the cells toward FBS used as a chemoattractant in the bottom of the well. Fewer 4T1-WNT5A cells migrated towards the chemoattractant than 4T1-vector cells indicating that migration was inhibited in the WNT5A expressing cells (Figure 1H). Likewise, addition of WNT5A containing conditioned media inhibited the level of migration in 4T1 cells when compared to cells treated with the control parental media (Figure 1H). Both 4T1 and 4T1-luc derived cells behaved similarly. In addition, similar results were obtained in another highly metastatic human cell line, MDA-231, engineered to over-express WNT5A (Figure S1).

**Figure 2:**
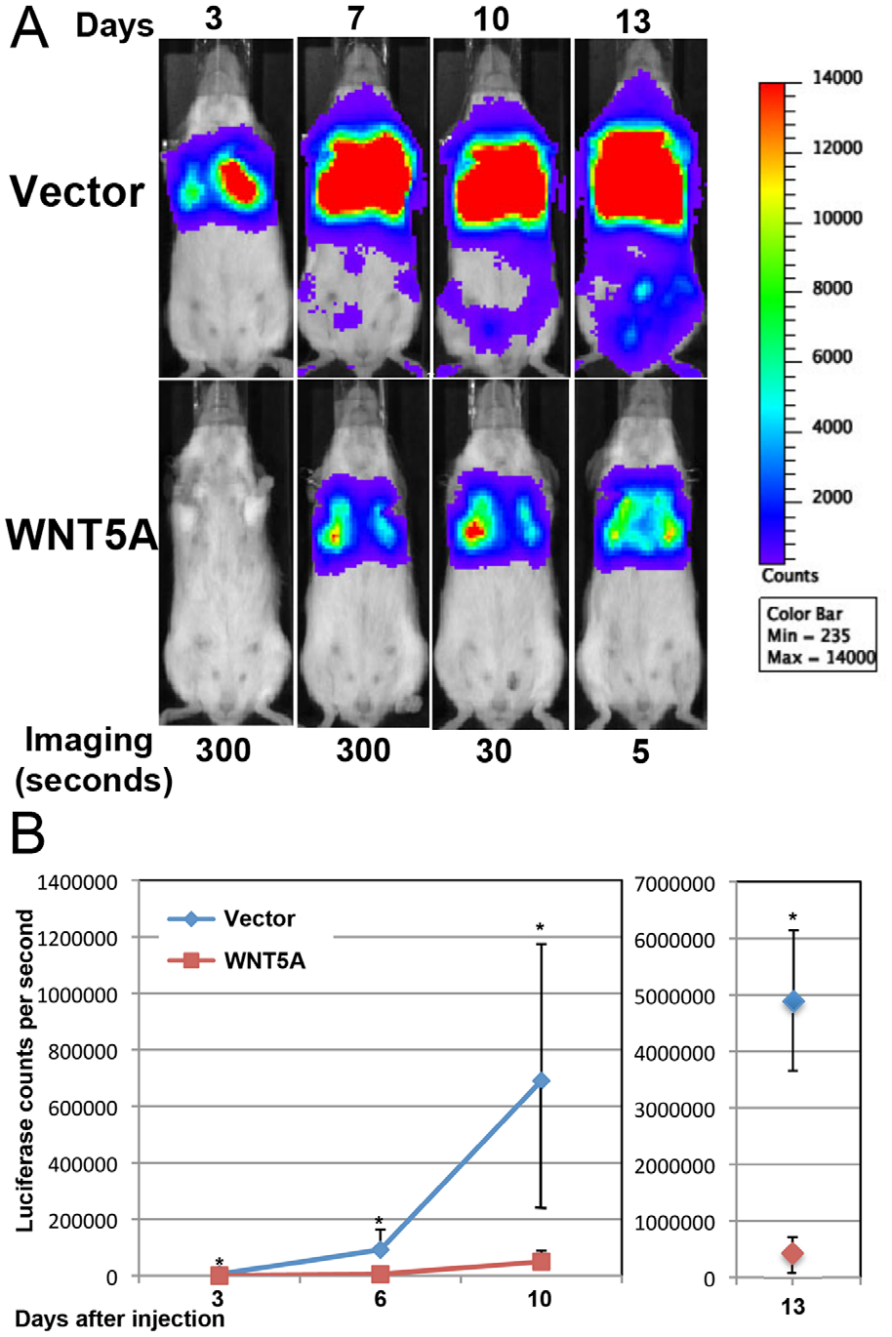
WNT5A inhibits metastasis to the lung. The tail vein assay was used to compare metastasis to the lung of vector only transduced 4T1-luc (Vector) and WNT5A expressing 4T1-luc (WNT5A) cells. (A) Luciferase imaging over several days after injection is shown. Red represents the highest intensity of luciferase signal. The imaging time in seconds is shown at the bottom. (B) The average and standard deviation from 6 mice in each group of luciferase counts per second is shown over time. Luciferase counts were significantly reduced in 4T1-Luc-WNT5A cells starting at 3 days after injection (*T-test p-value <0.05). Note Day 13 results are shown on a different scale.

Although migration plays a major role in metastasis, in vivo assays can measure other parameters of metastasis like invasion and colonization. 4T1-vector-luc and 4T1-WNT5A-luc cells were used in a tail-vein injection assay for metastasis (Figure 2). Equal numbers of vector or WNT5A expressing cells were injected into mouse tail veins (n = 6 each control and WNT5A). Luciferase was imaged at varying times after injection to monitor the location and growth of the cells in the lung (Figure 2A). Luciferase counts/second was graphed over time (Figure 2B). Cells expressing WNT5A had significantly lower signal in the lung suggesting reduced invasion and colonization in vivo.

### Global Analysis of Gene Expression

To begin to determine the molecular basis of WNT5A action in inhibiting migration and metastasis, we performed an Affymetrix microarray screen comparing gene expression in 4T1-WNT5A and 4T1-Vector cells. RNA was isolated from two separate passages of each cell line to generate two biological replicates. The high quality RNA was labeled and each sample was hybridized to a separate Affymetrix mouse 1.0 ST gene array. Results were analyzed using GeneSpring and Ingenuity Pathway Analysis Software. Ninety six genes were down-regulated at least two-fold (T-test, p<0.05) in WNT5A expressing cells relative to controls and twenty eight genes were up-regulated (Table S1).

RNA-seq has recently become a cost effective way to analyze global changes in gene expression [18], [19], [20]. In addition to determining relative levels of gene expression, the advantages of RNA-seq over microarray include: 1- Ability to identify novel or known splice variants; 2- Ability to identify expression of transcript variants at the single base level; and 3- Ability to assay novel genes or genes that are not present on the microarray chip. To further examine the effects of WNT5A on gene expression and transcript processing in the highly metastatic breast cancer cell lines, we performed RNA-seq analysis with the same RNA used in the microarray assay. After sequencing and alignment, 86.5% of the total reads from the vector control cells were aligned to the mouse reference genome and 84.4% from the WNT5A expressing cells were aligned (Table 1). A similar number of total genes were expressed in control and WNT5A cells (Table 2). Using Tophat/Cuffdiff analysis, 693 genes were identified as being significantly regulated by at least two-fold (FC2 q0.05) with 302 genes being up-regulated in WNT5A expressing cells and 391 being down-regulated (Table 2; Table S2). Over expression of the WNT5A transgene was confirmed in the sequencing analysis (Table S2).

**Table 1:** Sequence Alignment from CuffLinks.

	**Vector**	**Wnt5a**
Total Reads	31,604,816	43,743,888
Reads Removed	4,263,232 (13.5%)	6,821,966 (15.6%)
Reads Aligned to Reference Genome (mm9)	27,341,584 (86.5%)	36,921,922 (84.4%)

**Table 2:** Gene and Isoform Expression from CuffDiff.

Genes		
	Vector	Wnt5a
Total Genes Expressed	14,167	14,522
Vector Only	695	
Wnt5a Only		1,050
Up-regulated (>2-fold, q<0.05)		302
Down-regulated (<2-fold, q<0.05)		391
**Known Isoforms**		
	**Vector**	**Wnt5a**
Total Known Isoforms Expressed	17,105	17,687
Vector Only	1,322	
Wnt5a Only		1,904
Up-regulated (>2-fold, q<0.05)		562
Down-regulated (<2-fold, q<0.05)		595
**Novel Isoforms**		
	**Vector**	**Wnt5a**
Total Novel Isoforms Expressed	4,906	5,021
Vector Only	288	
Wnt5a Only		378
Up-regulated (>2-fold, q<0.05)		367
Down-regulated (<2-fold, q<0.05)		335

**Figure 3:**
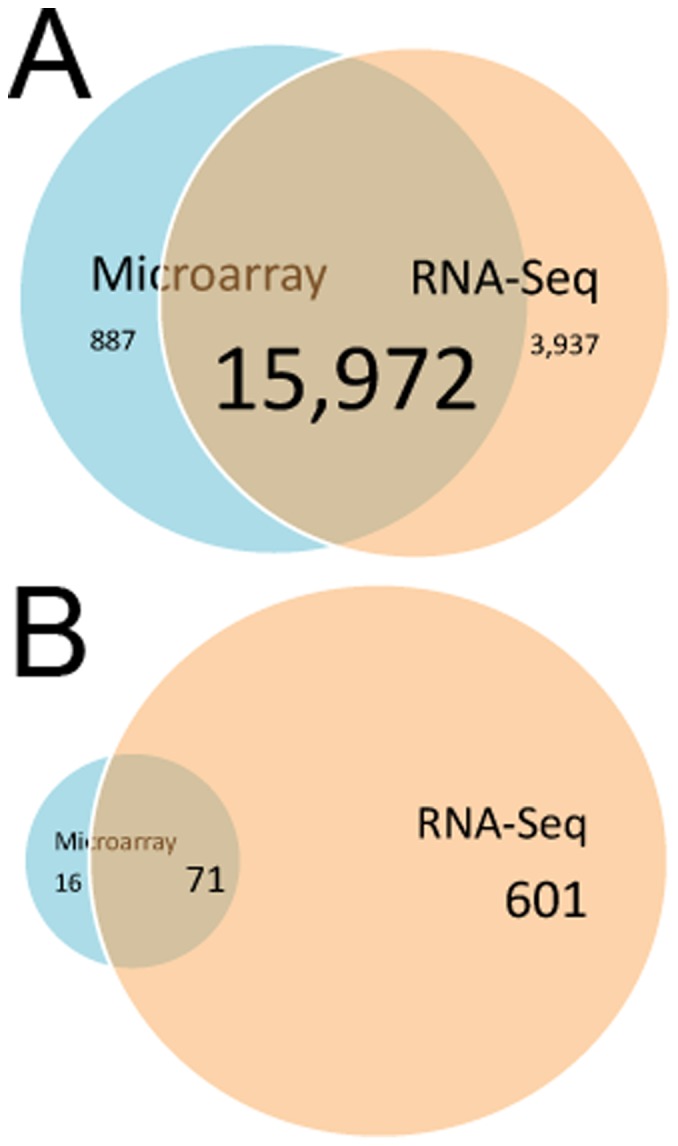
Comparison of Microarray and RNA-seq platforms. To compare data from the Microarray and RNA-Seq platforms, data from Genesprings (microarray) and DuffDiff (RNA-seq) were imported into Ingenuity Pathway Analysis Software. (A) Comparison of total gene expression in microarray and RNA-seq. For the microarray, the number of genes present after filtering for probe intensity between the 20^th^ and 100^th^ percentiles is shown in blue. The total number of reads aligned to the reference genome (FPKM>0) from the RNA-seq experiment is shown in pink. Most of the genes that are expressed overlap in both platforms; however, RNA-seq detected an overall larger number of expressed genes relative to the microarray (B) Comparison of regulated genes in microarray and RNA-seq experiments. All of the genes that were regulated up or down 2-fold, p<0.05 in the microarray are shown in blue. Genes that were determined to be regulated up or down 2-fold, q <0.05 in the RNA-seq experiment are shown in pink.

Overall gene expression from the microarray and RNA-seq experiments were compared (Figure 3). First, to compare the data, gene lists generated form Genesprings and CuffLinks had to be imported into Ingenuity software. This process resulted in elimination of additional genes tagged as duplicates. In the microarray, a total of 16,872 genes were listed as present in the control cells after filtering for expression within 20th to 100th percentiles, which is common since low signals are not reliable in the microarray. For RNA-Seq, a total of 19,991 genes were identified as being expressed in the control cells. This indicates that RNA-seq may be more sensitive in identifying expressed genes. Nevertheless, among the genes expressed the vast majority overlapped between the two platforms (Figure 3A). When looking at genes that were differentially expressed between control and WNT5A expressing cells, 87 genes were identified with the microarray platform while 672 were identified using RNA-seq (Figure 3B). Eighty –two percent of the genes identified as regulated using the microarray platform were also regulated in the RNA-seq analysis; however, more genes overall were found regulated using RNA-seq. This is likely due to the fact that RNA-seq can pick up genes that are expressed at a lower level and it can identify genes that might not be represented on the microarray. A few genes were identified as regulated in the microarray but not the RNA-seq. This could be due to cross hybridization of the RNA to closely related genes. In addition, consistent artifactual probe signals were recently identified in microarray screens using a variety of cell types [25].

**Figure 4:**
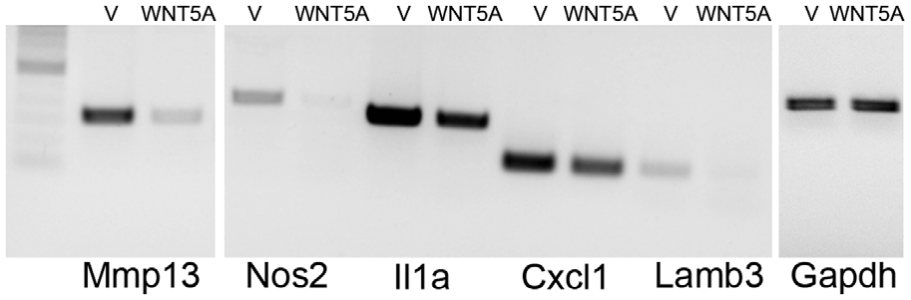
Expression of cell movement associated genes. Semi-quantitative RT-PCR was used to confirm regulation of a subset of cell movement associated genes in 4T1-WNT5A (WNT5A) relative to vector only control cells (V). Matrix metallopeptidase 13 (MMP13), nitric oxide synthase 2 (Nos2), interleukin 1, alpha (Il1a), chemokine (C-X-C motif) ligand 1 (Cxcl1), and laminin, beta 3 (Lamb3) were all down-regulated in WNT5A expressing cell. Gapdh was used as a normalization control. PCR products are shown in the linear range of product formation.

Pathway analysis indicated that genes associated with cell movement were highly regulated in WNT5A expressing cells in both the microarray assay (29/153 genes, p value 5.58×10^−8^ to 9.38×10^−3^; Table S3A) and the RNA-seq assay (125/744 genes; p value 7.13×10^−10^ to 5.74×10^−4^; Table S3B). Twenty-three of the 29 cell movement associated genes identified in the microarray assay were also identified in the RNA-seq assay (Table 3). Differential expression in the 4T1-WNT5A cells compared to the control 4T1-vector cells was confirmed for five selected genes (Mmp13, Nos2, Il1a, Cxcl1, and Lamb3) by semi-quantitative RT-PCR (Figure 4).

**Table 3:** Cell movement genes regulated in Wnt5a cells indentified in both microarray and RNA-Seq.

Gene Symbol	Gene Name	Fold Change Array	Fold Change RNA-Seq
AREG	amphiregulin	−2.25	−3.258
ARHGDIB	Rho GDP dissociation inhibitor (GDI) beta	−2.062	−2.661
CCK	cholecystokinin	−7.798	−24.312
CD38	CD38 molecule	2.513	4.744
CSF3	colony stimulating factor 3 (granulocyte)	−2.327	−2.476
CXCL1	chemokine (C-X-C motif) ligand 1	−2.119	−4.084
CXCL5	chemokine (C-X-C motif) ligand 6 (granulocyte chemotactic protein 2)	−2.523	−3.43
DAB2	disabled homolog 2	−2.073	−2.34
DLL1	delta-like 1 (Drosophila)	−2.728	−3.941
ENPP2	ectonucleotide pyrophosphatase/phosphodiesterase 2	−3.046	−3.968
IL1A	interleukin 1, alpha	−4.319	−4.078
ITGB7	integrin, beta 7	−2.064	−2.166
LAMA3	laminin, alpha 3	−2.153	−5.876
LAMB3	laminin, beta 3	−3.37	−4.4
LCP1	lymphocyte cytosolic protein 1 (L-plastin)	−2.097	−2.56
LY6A	lymphocyte antigen 6 complex, locus A	−2.1	−3.167
MMP10	matrix metallopeptidase 10 (stromelysin 2)	−2.74	−3.175
MMP13	matrix metallopeptidase 13 (collagenase 3)	−4.563	−5.204
NOS2	nitric oxide synthase 2, inducible	−4.574	−4.922
PGF	placental growth factor	−2.695	−3.131
SDC1	syndecan 1	−2.015	−2.001
SPP1	secreted phosphoprotein 1	−2.002	−2.807
STC1	stanniocalcin 1	−2.288	−3.254

### Differential Splicing in WNT5A Expressing Cells

**Figure 5:**
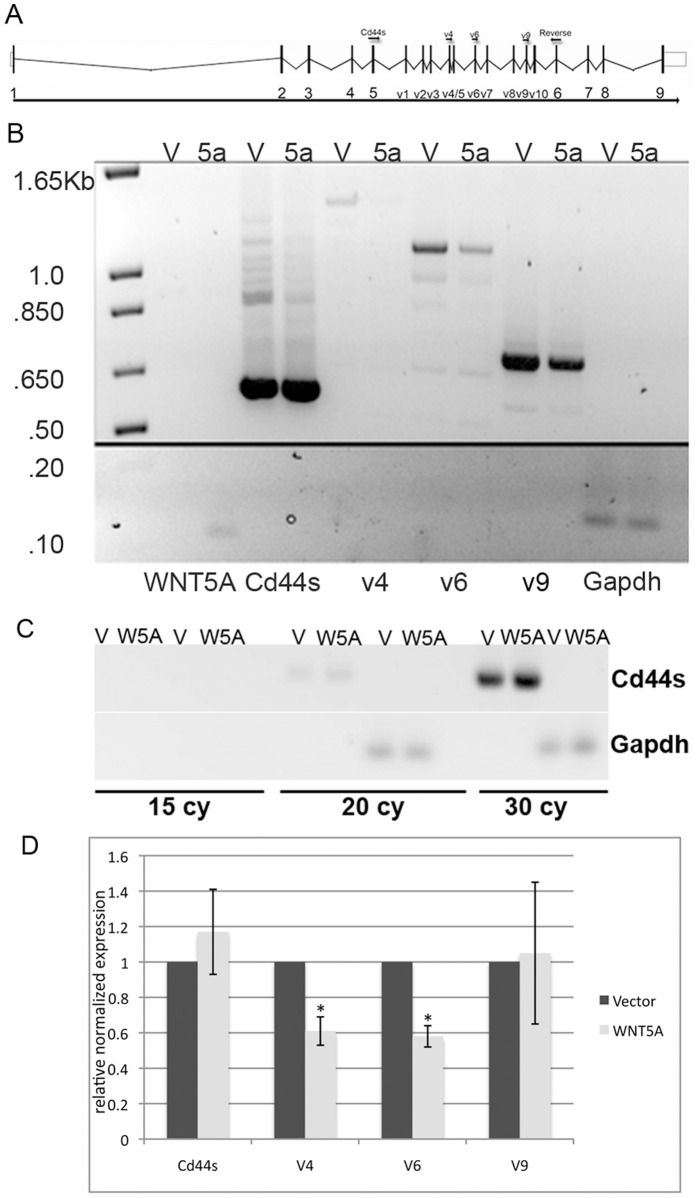
Alternative Cd44 exon usage. (A) Genomic structure of mouse Cd44 is shown. Cd44 has 9 non-variable exons numbered 1 to 9 (large). After non-variable exon 5 there are 10 variable exons numbered v1 to v10 (small). Primers used for RT-PCR are shown as small arrows on top of the genome structure. All PCR reactions used the same reverse primer in exon 6. Cd44s, the non-variable form of Cdd4, is the main PCR product amplified using the Cd44s primer in exon 5. Variable exons are amplified using primers in exon v4, v6 and v9. (B) RT-PCR for various Cd44 transcripts is shown (Cd44s, v4, v6, and v9). Expression of WNT5A was confirmed using primers to human WNT5A. Gapdh was used as a normalization control. (C) PCR was carried out for varying cycles to determine the linear range of product formation. Amplification of Cd44s is shown. Gapdh is used as a control for normalization. (D) Pixel density of bands from the images of the stained gels was determined and normalized to that of Gapdh. Bands in the linear range of product formation were used. Three separate experiments were analyzed. The vector was set to 1.0 and the relative levels of Cd44s, V4, V6 and V9 were determined. The average and standard deviation are shown on the graph. * = T-test p-value <0.05.

One of the advantages of RNA-seq is that differentially spliced transcripts can be identified from the sequencing alignment [19], [20]. We identified over 1000 transcripts that were significantly different in vector control and WNT5A cells using Cuffdiff 1.3.0. Transcripts from this list that were associated with cell movement are shown in Table 4. We then focused on Cd44 as an example of differences in exon usage between control and WNT5A expressing cells. Cd44 is an adhesion molecule with a complex genome structure [26]; Figure 5A). Mouse Cd44 has 10 variable exons denoted v1 through v10. Transcripts are subject to alternative splicing in this variable region, which predominantly affects the extracellular stem structure of the protein [26]. Variable exon usage has been shown to affect the adhesive properties of the protein and is associated with metastatic phenotype [27], [28]. Cd44s is used to designate the transcript without variable exons. Analysis of the sequence alignments indicated that most of the Cd44 mRNA in both control and WNT5A expressing cells was the non-variable form, Cd44s. Transcripts containing exons v1 through v3 were not detected in either control or WNT5A expressing cells; however, differential v4-v10 usage was suggested. To determine if there were differences in v4 to v10 usage, we performed RT-PCR using primers that would detect Cd44s as well as usage of exons v4, v6, and v9 (Figure 5; [23]. PCR product in the linear range of formation was characterized in three separate experiments (for example see Figure 5C). As suggested, Cd44s was highly expressed in both WNT5A and control cells (Figure 5B–D). In contrast, exon v4 usage was detected in control cells, whereas low v4 usage was detected in the WNT5A expressing cells (Figure 5B, D). Similar results were obtained for v5 (data not shown) and V6 (Figure 5 B, D). Significant differences in V9 expression were not detected in control and WNT5A expressing cells (Figure 5B, D). The results suggest that expression of WNT5A in highly metastatic cells results in reduced expression of specific variant Cd44 transcripts.

**Table 4:** Differential exon usage for cell movement associated genes in Wnt5a compared to vector control cells.

Gene Symbol	Gene Name	sqrt(JS)
ADRBK1	adrenergic, beta, receptor kinase 1	0.176485
APP	amyloid beta (A4) precursor protein	0.2694
ARTN	artemin	0.305967
AXL	AXL receptor tyrosine kinase	0.349732
CD40	CD40 molecule, TNF receptor superfamily member 5	0.406921
CD44	CD44 molecule (Indian blood group)	0.092575
CDK5	cyclin-dependent kinase 5	0.193261
CTBP1	C-terminal binding protein 1	0.17588
CTBP2	C-terminal binding protein 2	0.226954
CTSB	cathepsin B	0.0370863
EPHB2	EPH receptor B2	0.311454
ETV4	ets variant 4	0.296399
GNA12	guanine nucleotide binding protein (G protein) alpha 12	0.110352
IL17RA	interleukin 17 receptor A	0.237452
IL6R	interleukin 6 receptor	0.44103
JUP	junction plakoglobin	0.105648
KIF1C	kinesin family member 1C	0.185524
KIF20B	kinesin family member 20B	0.241796
LTBP2	latent transforming growth factor beta binding protein 2	0.122796
MAP4K4	mitogen-activated protein kinase kinase kinase kinase 4	0.230163
MAPK7	mitogen-activated protein kinase 7	0.165228
MAVS	mitochondrial antiviral signaling protein	0.184029
MEN1	multiple endocrine neoplasia I	0.358877
NDST1	N-deacetylase/N-sulfotransferase (heparan glucosaminyl) 1	0.215596
NFAT5	nuclear factor of activated T-cells 5, tonicity-responsive	0.317092
PTPRA	protein tyrosine phosphatase, receptor type, A	0.0952815
RAP1GAP	RAP1 GTPase activating protein	0.414666
ROCK1	Rho-associated, coiled-coil containing protein kinase 1	0.29537
RUNX2	runt-related transcription factor 2	0.445803
SRC	v-src sarcoma (Schmidt-Ruppin A-2) viral oncogene homolog (avian)	0.0775897
STAT3	signal transducer and activator of transcription 3 (acute-phase response factor)	0.243735
STK38L	serine/threonine kinase 38 like	0.252149
TGFBR1	transforming growth factor, beta receptor 1	0.22866
TNFAIP3	tumor necrosis factor, alpha-induced protein 3	0.329969
TNFRSF1B	tumor necrosis factor receptor superfamily, member 1B	0.0822495
UNC5B	unc-5 homolog B (C. elegans)	0.180276
WISP1	WNT1 inducible signaling pathway protein 1	0.23867
WWTR1	WW domain containing transcription regulator 1	0.114948

## Discussion

To characterize the effects of WNT5A on metastatic behavior and to begin to determine mechanisms of WNT5A action in metastasis, we generated WNT5A expressing 4T1 and MBA-MD-231 cell lines. Expression of WNT5A in the normally highly metastatic cells reduced metastatic behavior of the cells including migration and lung colonization after tail vein injection. Global analysis of gene expression indicated down-regulation of a number of migration associated pathways. Furthermore, we identified alternative splicing of migration-associated genes including Cd44. The expression of specific variant isoforms of Cd44 was reduced in WNT5A expressing cells.

Several previous studies have addressed the effects of WNT5A on the progression of invasive breast cancer. In human breast cancers, loss of Wnt-5a protein is associated with poor outcome in part because of an increase in distant metastases, suggesting a suppressive role of WNT5A in breast cancer metastasis [8], [9], [10]. Previous studies showed that WNT5A could inhibit migration in various non-malignant and malignant cells lines [16], [29]. Here we show that mis-expression of WNT5A in the highly metastatic breast cancer cell lines, 4T1 and MDA-MB-231, inhibits migration and metastasis as measured by transwell and a tail vein injection assays consistent with previous reports [16]. Post-translational protein targets of WNT5A in breast cancers have also been identified [14], [15], [30] however, a global comparison of gene expression in WNT5A low and high expressing breast cancer cells has not been reported. Here we compared gene expression in control and WNT5A expressing 4T1 cells by Affymetrix microarray and RNA-seq assays. Inhibition of cell migration characterized here is likely an autocrine signaling event. It has been shown that Wnt5a can have paracrine effects on the tumor microenvironment that can impact breast cancer progression [11]. For example, WNT5A can induce MMP expression in tumor-associated macrophages thereby promoting invasion [11]. This report focuses only on the autocrine effects of WNT5A on tumor cells and does not address any potential paracrine effects of WNT5A on the tumor microenvironment.

Previous studies have identified gene expression signatures that define breast cancer metastasis [31]. Microarray and RNA-seq performed in this report provide a comprehensive view of changes in gene expression elicited by over-expression of WNT5A in metastatic breast cancer cells. We identified several potential targets of WNT5A that are associated with cell migration including chemokines, Cxcl1 and Il1a, ECM associated proteins, Mmp13 and Lamb3, as well as an inflammation responsive enzyme, Nos2. All of these genes were down-regulated in WNT5A expressing cells and have known roles in tumor progression. Cxcl1has an important role in promoting breast cancer metastasis and, recently, was shown to link metastasis to drug resistance [32]. Polymorphisms in Il1a are associated with increased breast cancer risk [33]. Many studies have implicated Mmps in promoting metastasis. Recently, a selective inhibitor of Mmp13 was shown to delay the onset of tumor associated osteolytic lesions in a model of bone metastasis; however, no effects on soft organ metastasis were observed [34]. Lamb3 is the beta chain for laminin 5, which has been shown to promote migration of breast cancer cells [35]. Increased inducible Nos2 was associated with poor survival in estrogen receptor-negative breast cancer patients [36]. Down-regulation of any of these genes by WNT5A would be expected to contribute to inhibition of tumor progression. The list of differentially expressed genes provides information to guide future mechanistic studies aimed at determining how WNT5A affects tumor progression and metastasis.

The signaling cascade initiated by WNT5A that regulates migration in breast cancer is not clear and may be context specific [13]. WNT5A has been shown to act through numerous ß-catenin independent signaling pathways to influence migration in a cell type specific manner. Pathways activated by WNT5A include G-protein coupled receptor pathways acting through Protein Kinase A and cAMP Responsive Element Binding protein as well as through Calcium dependent pathways like Casein Kinase I [24], [30]. Protein Kinase C and Rho activation have also been implicated in WNT5A mediated cell movement and cytoskeletal organization [37], [38]. WNT5A can also act to antagonize canonical ß-catenin dependent signaling and several distinct cell type specific mechanisms for this antagonism have been elucidated including calcium dependent activation of NEMO-like kinase, an inhibitor of the TCF/LEF transcriptional complex [13], [37], [38]. Any of these pathways or yet unidentified pathways could lead to down-regulation of the migration-associated genes identified above.

Alternative splicing programs that can identify various breast cancer subtypes have been identified [39]. In addition, epithelial to mesenchymal transition (EMT) induced by overexpression of Twist in normal breast cells promotes a program of alternative splicing associated with metastasis [40]. Here we identify several alternatively spliced genes in WNT5A expressing cells that are associated with cell migration. One of the genes identified was Cd44. It has been shown that CD44 isoform switching is involved in EMT and metastasis [27]. We show reduced levels of variant Cd44 isoforms in WNT5A expressing cells. This would fit with the reduced level of lung colonization observed in the WNT5A expressing cells. Recently, it was shown that variant forms of CD44 are induced by the splicing factor Esrp1, which can also enhance lung colonization of metastatic 4T1 cells [28]. In this case, Esrp1 expression was independent of EMT-related mechanisms since there was no difference in the expression of EMT markers in Ersp1 high and low cells [28]. The authors suggested Esrp1 was regulated by other mechanisms. Down-regulation of Esrp1 by WNT5A would be expected to result in less Cd44v and less lung metastasis, which is what we observed. Mining our RNA-seq data indicated that Esrp1 was down-regulated in WNT5A-expressing cells but it did not pass our False Discovery Rate (q-value) cut-off of significance. Regulation of additional splicing-associated genes was identified through mining of the RNA-seq and microarray data (Table S4). In some cases, splicing genes were themselves differentially spliced in WNT5A-4T1 cells. For example, a long transcript variant of Rbfox2 (NM_053104) was up-regulated in Wnt-5a-4T1 cells whereas the short variant (NM_001110830) was down-regulated. Rbfox2 has been shown to be critical in regulating cancer subtype specific alternative splicing programs [39] and in mediating EMT specific splicing programs [40]. With regards to WNT5A signaling, the functional consequences of differential expression of Rbfox2 transcripts is not clear, but future studies will address these issues.

## Supporting Information

Figure S1 **Generation and characterization of Wnt5 expressing MDA-MB-231 cells.** (A) Western blot was used to show expression of the WNT5A transgene in MDA-MB-231 cells (231) relative to vector transduced (V) and WNT5A transduced cells. (B) A transwell migration assay indicated that WNT5A expressing MDA-MD-231 cells had reduced migration toward serum free media (SFM), 10% FBS and 20% FBS relative to vector only control cells. (C) The transwell migration assay was used to determine the effects of WNT5A conditioned media (WNT5A CM) on cell migration towards 10% FBS. Treatment of cells with WNT5A conditioned medium inhibited cell migration. (D) MDA-MB-231 cells were placed in SFM in the top well of the transwell while either parental or WNT5A conditioned media was placed in the bottom well. Migration towards WNT5A CM was inhibited (D, left). Migration was also inhibited if conditioned media was placed in both top and bottom chambers (D, right). * = T-test p-value <0.05, **p<0.01, ***p<0.001.
(TIF)

Table S1 (XLS)

Table S2 (XLSX)

Table S3 (XLS)

Table S4 (XLS)
