# Supplementary figures and images for: WNT5A Inhibits Metastasis and Alters Splicing of Cd44 in Breast Cancer Cells
Source: PLoS One. 2013 Mar 6;8(3):e58329. doi: 10.1371/journal.pone.0058329 (PMC3590134; doi:10.1371/journal.pone.0058329)

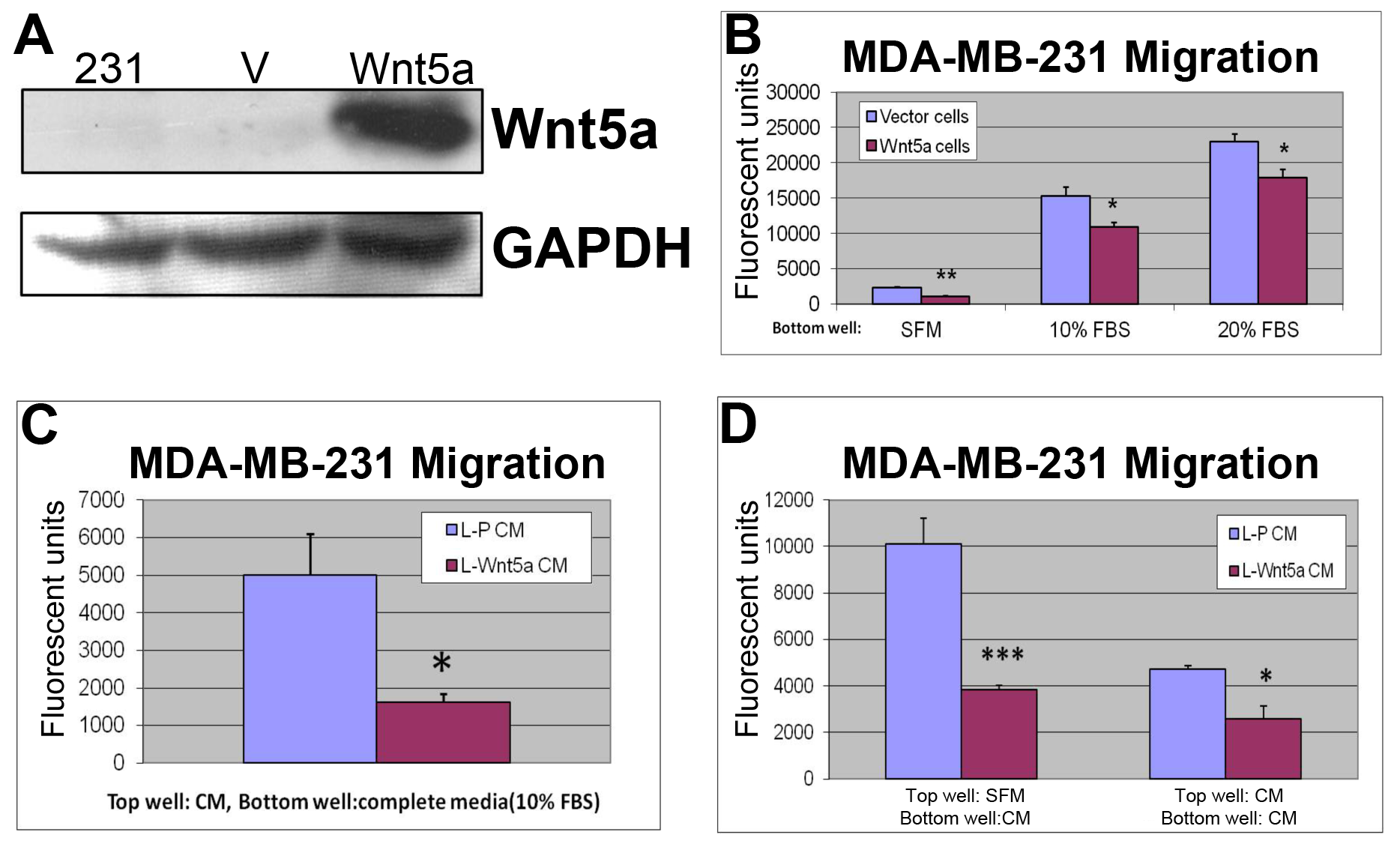

Supplement: Figure S1 — Generation and characterization of Wnt5 expressing MDA-MB-231 cells. (A) Western blot was used to show expression of the WNT5A transgene in MDA-MB-231 cells (231) relative to vector transduced (V) and WNT5A transduced cells. (B) A transwell migration assay indicated that WNT5A expressing MDA-MD-231 cells had reduced migration toward serum free media (SFM), 10% FBS and 20% FBS relative to vector only control cells. (C) The transwell migration assay was used to determine the effects of WNT5A conditioned media (WNT5A CM) on cell migration towards 10% FBS. Treatment of cells with WNT5A conditioned medium inhibited cell migration. (D) MDA-MB-231 cells were placed in SFM in the top well of the transwell while either parental or WNT5A conditioned media was placed in the bottom well. Migration towards WNT5A CM was inhibited (D, left). Migration was also inhibited if conditioned media was placed in both top and bottom chambers (D, right). * = T-test p-value <0.05, **p<0.01, ***p<0.001. (TIF) [file pone.0058329.s001.tif]
